# Laparoscopic Distal Pancreatectomy with Splenectomy for a Multiloculated Primary Splenic Epidermoid Cyst Mimicking a Lymphangioma in an Older Woman: A Case Report

**DOI:** 10.70352/scrj.cr.26-0466

**Published:** 2026-10-24

**Authors:** Ryotaro Hashizume, Hiroshi Imai, Tetsuya Murata, Yasuo Okura

**Affiliations:** 1 Department of Pathology and Matrix Biology, Mie University Graduate School of Medicine, Tsu, Mie, Japan; 2 Department of Genomic Medicine, Mie University Hospital, Tsu, Mie, Japan; 3 Pathology Division, Mie University Hospital, Tsu, Mie, Japan; 4 Department of Pathology and Laboratory Medicine, JA Suzuka General Hospital, Suzuka, Mie, Japan; 5 Department of Surgery, Suzuka Kaisei Hospital, Suzuka, Mie, Japan

**Keywords:** epidermoid cyst, spleen, lymphangioma, immunohistochemistry, distal pancreatectomy, pancreatic fistula

## Abstract

**INTRODUCTION:**

Primary splenic epidermoid cysts typically occur in young adults as unilocular lesions and are usually distinguished from lymphangiomas on histopathology, although preoperative imaging differentiation remains challenging. A multiloculated presentation in an older patient is uncommon and can be mistaken for a lymphangioma.

**CASE PRESENTATION:**

Herein, we report a 75-year-old asymptomatic woman with a multiloculated splenic cyst that was managed conservatively for approximately 10 years. CT and MRI favored a lymphangioma, and surgical resection was performed because of interval growth. Laparoscopic distal pancreatectomy with splenectomy (DP-S) was required because the cyst was firmly adherent to the pancreas. Histopathological examination revealed a 3.7 × 3.1-cm multiloculated cyst within the splenic capsule, lined by stratified squamous epithelium and associated with foamy histiocytes and cholesterol crystal clefts. Immunohistochemistry demonstrated negativity for CD31, CD34, and D2-40, and positivity for AE1/AE3, cytokeratin (CK) 5/6, and CK7, confirming a primary splenic epidermoid cyst. Negativity for calretinin and Wilms tumor 1 (WT-1) excluded a mesothelial cyst, whereas basal p63 positivity and carbohydrate antigen 19-9 expression supported the multilayered subtype. A postoperative pancreatic fistula was managed conservatively, and the patient was discharged on POD 34.

**CONCLUSIONS:**

Integrated clinicopathological assessment, rather than any single marker, is needed to distinguish a multiloculated splenic epidermoid cyst from a lymphangioma; comprehensive immunohistochemistry, including D2-40, calretinin, WT-1, and p63, is a useful adjunct. This case also shows that a benign splenic cyst may require distal pancreatectomy: when dense pancreatic adhesion is encountered intraoperatively, en bloc DP-S can become an unavoidable step toward safe and complete resection, and postoperative pancreatic fistula remains a relevant complication to anticipate.

## Abbreviations

CA19-9
: carbohydrate antigen 19-9
CEA
: carcinoembryonic antigen
CK
: cytokeratin
DP-S
: distal pancreatectomy with splenectomy
WT-1
: Wilms tumor 1

## INTRODUCTION

Splenic cysts are rare, with an autopsy prevalence of 0.07%–0.13%, and are classified as either parasitic or nonparasitic. Epidermoid cysts are typically solitary and occur more commonly in younger patients.^1)^ Splenic lymphangiomas usually present as multilocular, thin-walled cystic lesions that overlap with epidermoid cysts on imaging, often requiring histopathological examination for diagnosis.^2)^ When such lesions are adherent to the pancreatic tail, differentiating them from a pancreatic cystic neoplasm becomes an additional diagnostic challenge^3)^ and may necessitate combined resection.^4)^

We report a multiloculated primary splenic epidermoid cyst in a 75-year-old woman that was managed conservatively for nearly a decade as a presumed lymphangioma, in whom laparoscopic splenectomy was planned for documented interval growth and diagnostic uncertainty but was extended to en bloc DP-S because of dense intraoperative pancreatic adhesion.

## CASE PRESENTATION

A 75-year-old asymptomatic woman was referred for evaluation of a known splenic mass. The lesion had been identified incidentally 10 years earlier and managed conservatively under the presumptive diagnosis of lymphangioma. It was not reimaged for approximately 7 years thereafter, and reassessment was prompted by an unrelated finding during a routine health check. Her medical history included hypertension and dyslipidemia, without abdominal trauma or parasitic exposure.

Physical examination was unremarkable. Laboratory evaluation, including a complete blood count, liver function tests, and serum amylase (68 IU/L), was normal except for a mildly reduced estimated glomerular filtration rate (49 mL/min/1.73 m2) and borderline glycemic control (hemoglobin A1c, 6.3%). Preoperative tumor markers were not assessed because the lesion was presumed to be a lymphangioma. Serum CEA (3.8 ng/mL) and CA19-9 (12.4 U/mL), measured 10 years earlier, were within normal limits.

Contrast-enhanced CT performed 4 months before surgery demonstrated a well-circumscribed, predominantly hypoattenuating, multiloculated cystic mass measuring 4.4 × 3.2 cm at the splenic hilum. The mass contained thin septa with faint enhancement and showed no radiological evidence of pancreatic invasion (**Fig. 1A** and **1B**). T2-weighted MRI performed 2 years and 4 months before surgery showed a hyperintense, multiloculated lesion without diffusion restriction (**Fig. 1C** and **1D**). Review of serial imaging showed that the lesion had enlarged from 1.9 cm in maximum diameter approximately 10 years before surgery to 4.2 cm about 2 years before surgery—with no interval imaging between these 2 time points—and the lesion had remained stable thereafter (**Fig. 1E**). Because of this documented growth together with persistent diagnostic uncertainty, resection was discussed. Continued observation was also offered, and the patient elected to undergo surgery, which was scheduled after her retirement.

**Fig. 1:**
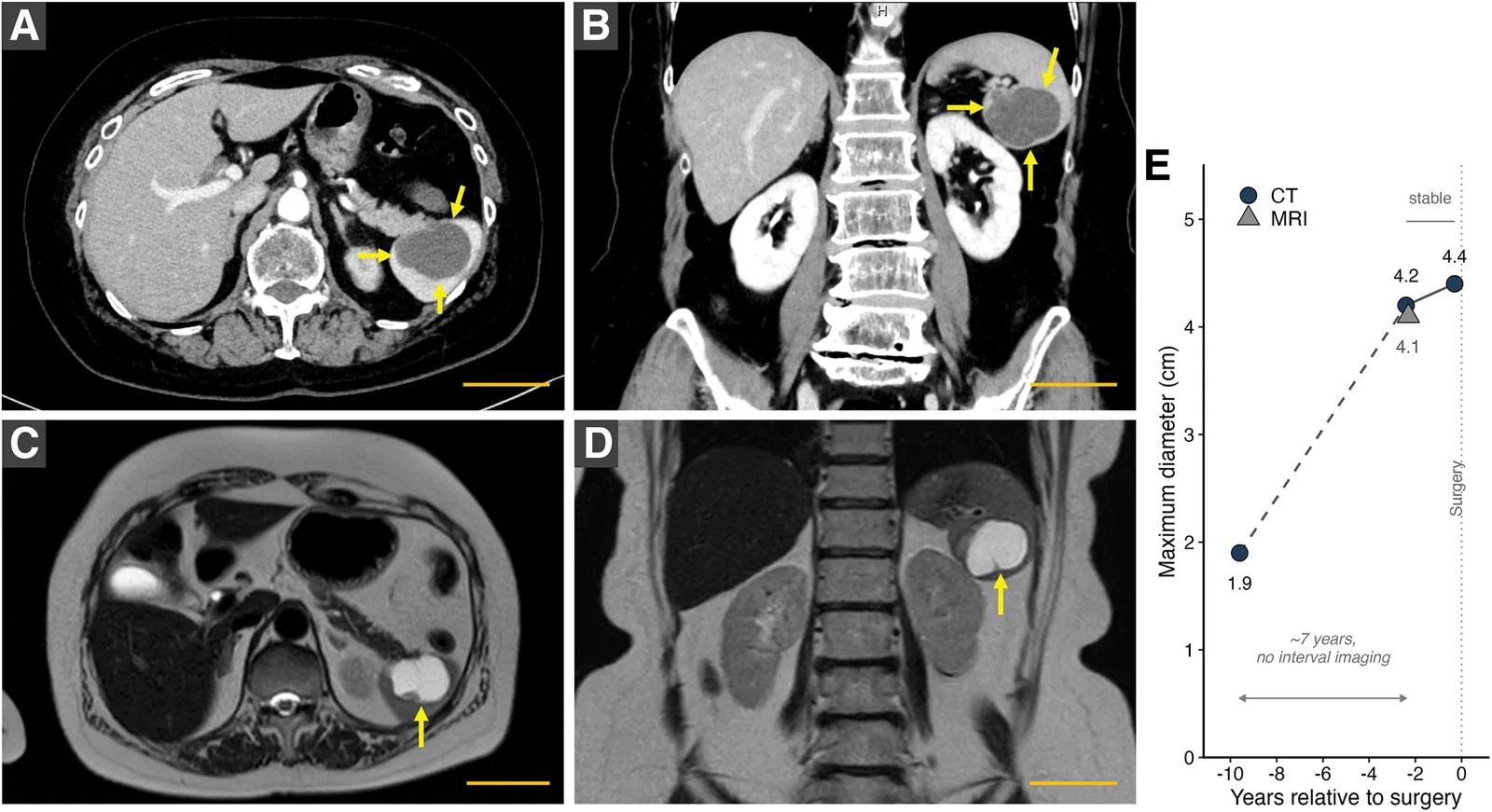
Preoperative imaging and growth of the splenic lesion. (**A**) Contrast-enhanced CT, axial view, and (**B**) coronal reconstruction, obtained 4 months before surgery, showing a well-circumscribed, predominantly hypoattenuating cystic mass (arrows) at the splenic hilum. (**C**) Axial and (**D**) coronal T2-weighted magnetic resonance images obtained 2 years and 4 months before surgery, showing the lesion (arrows) as a markedly hyperintense multiloculated cystic mass with thin internal septa. (**E**) Serial maximum diameter of the lesion on cross-sectional imaging relative to the time of surgery (CT, circles; MRI, triangle). The lesion enlarged from 1.9 to 4.2 cm over the preceding years and remained stable thereafter (4.4 cm shortly before surgery); no imaging was available during the intervening interval (dashed line). Scale bars (**A**–**D**), 5 cm.

Laparoscopic splenectomy was planned. Under general anesthesia, with the patient placed in the right semi-lateral decubitus position, a 12-mm umbilical camera port, two 5-mm working ports, and a 10-mm left flank port were placed. After mobilization of the splenic flexure, the splenocolic, splenorenal, and gastrosplenic ligaments and the short gastric vessels were divided, and the phrenicosplenic ligament was then incised to complete splenic mobilization.

Intraoperatively, the cyst was densely adherent to the pancreatic body and tail with no identifiable dissection plane; the procedure was therefore extended to an en bloc DP-S. Because the pancreatic parenchyma was thick (15 mm on preoperative imaging), it was gradually compressed for 5 minutes before transection, which was performed with 2 firings of a reinforced linear stapler (Tri-Staple 2.0 reinforced reload, 60 mm, black; Medtronic, Dublin, Ireland). The specimen was retrieved through an extended umbilical incision, and a closed-suction drain was placed in the left subphrenic space. The operative time was 285 minutes, and the estimated blood loss was 30 mL.

The postoperative course was complicated by a pancreatic fistula. Drain amylase levels were 6764 IU/L on POD 1 and 3418 IU/L on POD 3, exceeding 3 times the upper limit of the serum reference range. The fistula was classified as grade B according to the International Study Group of Pancreatic Surgery (ISGPS) definition.^5)^ Continuous intravenous octreotide (300 µg/day) was administered from PODs 1 to 4. A low-grade fever associated with a peripancreatic fluid collection was treated with cefmetazole, and the drain was managed by exchange and periodic irrigation. Oral intake resumed on POD 3, the drain was removed on POD 25, and the patient was discharged on POD 34 without reoperation.

Macroscopically, the en bloc specimen comprised the distal pancreas and spleen (**Fig. 2A**). Serial sectioning revealed a 3.7 × 3.1-cm multiloculated splenic cyst with loculi of varying sizes containing yellowish keratinous material and translucent fluid (**Fig. 2B**). All cystic components were confined within the splenic capsule, and the adjacent pancreatic parenchyma was unremarkable.

**Fig. 2:**
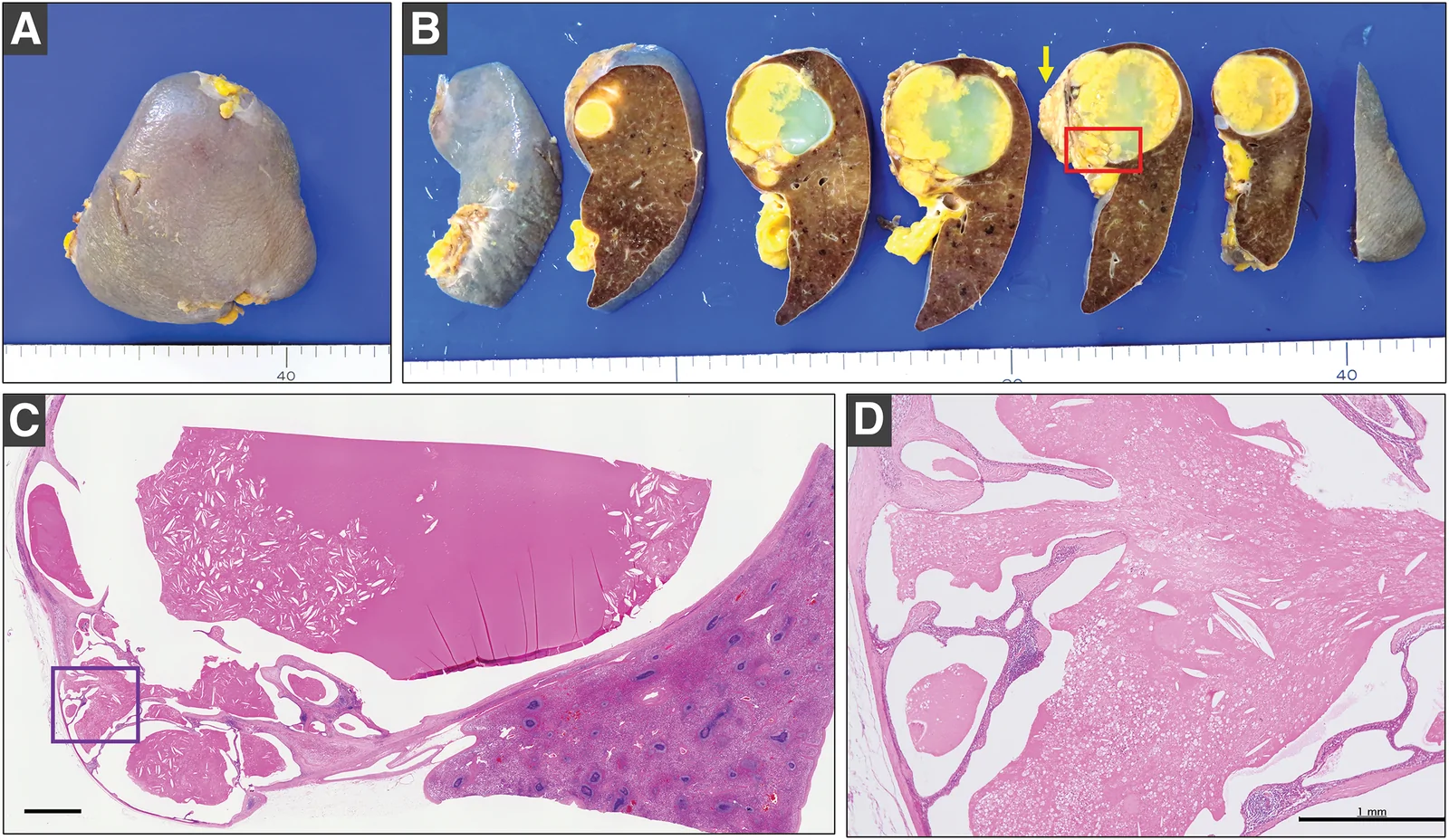
Gross and microscopic findings of the resected specimen. (**A**) External view of the en bloc distal pancreas and spleen specimen. (**B**) Serial cross-sections showing a multiloculated cyst with loculi of varying sizes containing yellowish keratinous material and translucent fluid. The yellow arrow indicates pancreatic tail tissue resected en bloc with the spleen, and the red square indicates the region examined microscopically in panel (**C**). (**C**) Low-power H&E staining showing eosinophilic keratinous content with cholesterol crystal clefts bordered by a thin fibrous wall and splenic parenchyma; the violet square indicates the region magnified in panel (**D**) (scale bar, 2 mm). (**D**) Higher magnification of the peripheral multiloculated region showing smaller cystic spaces and cholesterol crystal clefts within eosinophilic material (scale bar, 1 mm). H&E; hematoxylin and eosin

Histologically, the cyst lumen contained eosinophilic keratinous debris with abundant cholesterol crystal clefts, while the periphery exhibited smaller loculi (**Fig. 2C**, **2D**). The cyst was lined by attenuated, stratified squamous-like epithelium, 1 to 3 cell layers thick, without cytologic atypia, and was accompanied by foamy histiocytes (**Fig. 3A** and **3B**). Immunohistochemistry demonstrated negativity for CD31, CD34, and D2-40, and positivity for AE1/AE3, CK 7, and CK5/6, with negativity for CK20 (**Fig. 3C**–**3I**). The lining was negative for calretinin and WT-1, arguing against a mesothelial origin, and negative for CEA, whereas CA19-9 was positive and p63 showed nuclear positivity in the basal cell layer (**Fig. 3J**–**3O**). These findings supported the diagnosis of a primary splenic epidermoid cyst of the multilayered subtype and excluded lymphatic, vascular, and mesothelial origins.

**Fig. 3:**
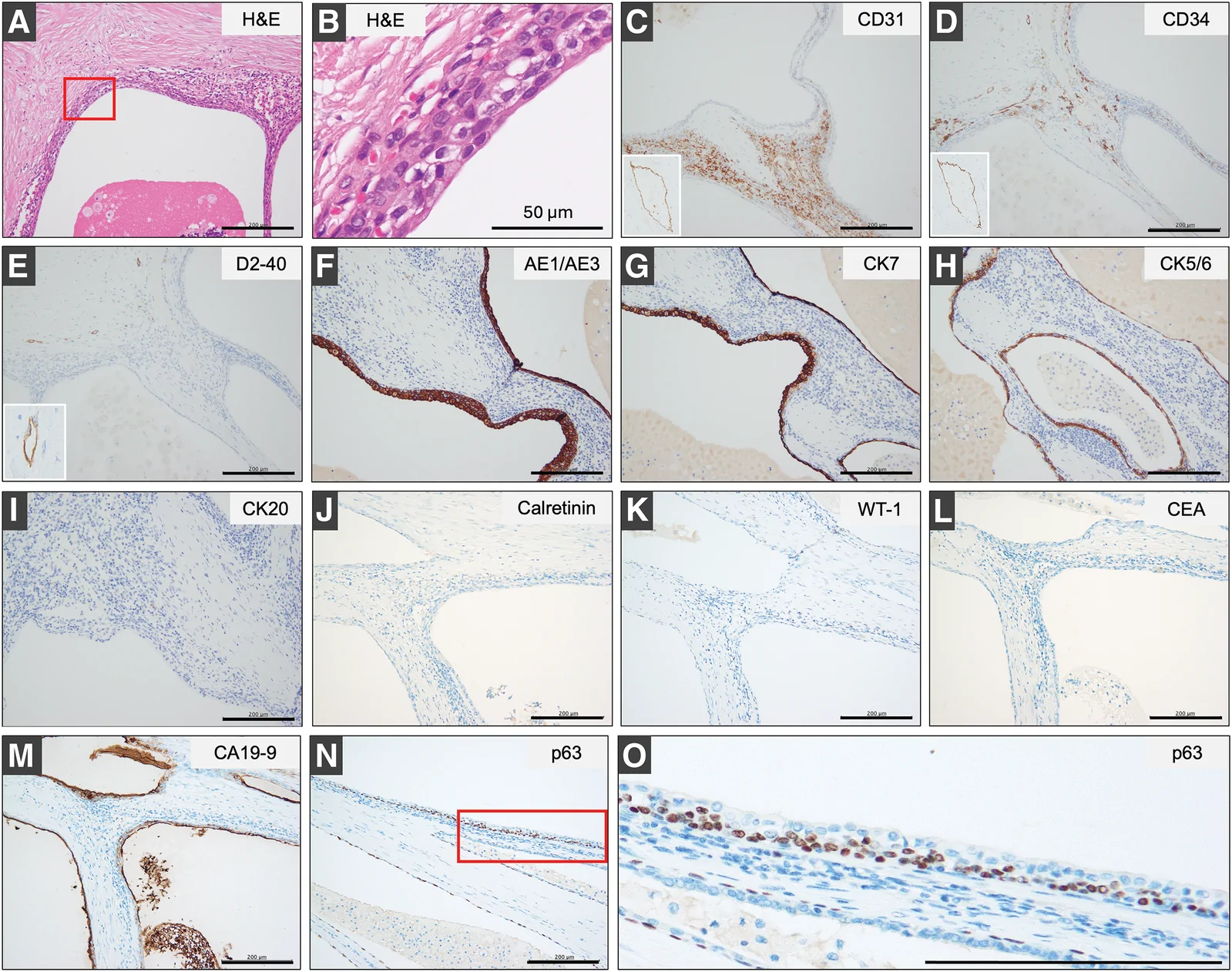
Histological and immunohistochemical characterization of the cyst lining. (**A**) H&E staining showing the attenuated cyst lining overlying keratinous contents; the red square indicates the region magnified in panel (**B**). (**B**) Higher magnification (H&E) showing stratified squamous-like epithelium 1 to 3 cell layers thick, without cytologic atypia. (**C**) CD31, (**D**) CD34, and (**E**) D2-40: the cyst lining is negative for all 3 markers, whereas adjacent vascular and lymphatic endothelia serve as internal positive controls (insets). (**F**) AE1/AE3, (**G**) CK7, and (**H**) CK5/6: the lining is diffusely positive. (**I**) CK20, (**J**) calretinin, (**K**) WT-1, and (**L**) CEA: the lining is negative. (**M**) CA19-9: the lining is positive. **(N)** p63: nuclear positivity is present in the basal cell layer; the red square indicates the region magnified in panel (**O**). (**O**) Higher magnification of p63, showing basal nuclear staining. Scale bars, 200 µm (**A, C**–**O**); 50 µm (**B**). CA19-9, carbohydrate antigen 19-9; CEA, carcinoembryonic antigen; CK, cytokeratin; H&E, hematoxylin and eosin; WT-1, Wilms tumor 1

At 5 weeks postoperatively, the patient remained well, without recurrence or further complications.

## DISCUSSION

This case raises several instructive points that span diagnosis and surgical management.

First, multiloculated splenic epidermoid cysts can closely mimic lymphangiomas on cross-sectional imaging, and this mimicry has been reported previously.^6)^ In our patient, serial CT and MRI over several years consistently favored lymphangioma, and the diagnosis was established only after resection. The differential diagnosis of a multiloculated splenic cyst includes lymphangioma, mesothelial cyst, pseudocyst, and, when the lesion abuts the pancreatic tail, a pancreatic cystic neoplasm.^1,3)^ Rather than relying on any single marker, we based the diagnosis on the integration of morphology and a panel of immunostains. The presence of a true epithelial lining—an attenuated, stratified, squamous-like epithelium positive for AE1/AE3, CK5/6, and CK7 and negative for CK20—argued against a pseudocyst, which by definition lacks an epithelial lining. D2-40 (podoplanin) marks the lymphatic endothelium of splenic lymphangiomas^7)^; its absence in the cyst lining, together with negativity for CD31 and CD34, excluded a lymphatic or vascular origin. A mesothelial cyst was excluded by negativity for calretinin and WT-1. Finally, confinement of the entire cystic lesion within the splenic capsule, together with a squamous rather than mucinous columnar lining, argued against a pancreatic cystic neoplasm.

Second, the immunophenotype is consistent with established descriptions of splenic epidermoid cysts. Hirabayashi et al. reported that both histological subtypes express CK5/6 and CK7 and lack CK20, whereas p63, CEA, CA19-9, and basal D2-40 expression—present in approximately 60% of cases—are confined to the multilayered subtype.^8)^ In our case, the stratified squamous-like lining corresponds morphologically to the multilayered subtype, and this classification is supported immunohistochemically by nuclear p63 expression in the basal cell layer and by CA19-9 positivity, both of which are features of that subtype. Negativity for CEA and the absence of basal D2-40 expression do not argue against this classification, because these markers are variably expressed even within the multilayered subtype. Because the lining is predominantly squamous with focal CK7 and CA19-9 expression rather than discrete glandular structures, we describe it as a squamous epithelial lining with focal glandular-type marker expression rather than overt squamous-glandular differentiation. Foamy histiocytes and cholesterol clefts likely reflect the chronic accumulation of keratinous debris.

Third, and most relevant to surgical practice, this case demonstrates why a benign splenic cyst may nonetheless require distal pancreatectomy. The indication for surgery was not size alone but the combination of documented long-term growth—the lesion enlarged from 1.9 to 4.4 cm over approximately a decade, although imaging was not obtained throughout this interval, and stabilized over the final 2 years (**Fig. 1E**)—persistent diagnostic uncertainty, and the patient’s informed preference for resection after shared decision-making that included the option of continued observation. Notably, the planned procedure was laparoscopic splenectomy, and preoperative imaging did not show pancreatic invasion. Intraoperatively, however, the cyst was densely adherent to the pancreatic body and tail with no cleavage plane, and en bloc distal pancreatectomy was performed as an unplanned but unavoidable step toward safe and complete resection. A comparable course—a benign cyst so adherent to adjacent structures that en bloc DP-S became necessary—has been reported for a pancreatic lymphoepithelial cyst.^4)^ This underscores that dense inflammatory adhesion, rather than malignancy, can dictate the extent of resection.

Finally, the principal morbidity was a postoperative pancreatic fistula (ISGPS grade B), which resolved with conservative management. Clinically relevant pancreatic fistula complicates approximately 20%–30% of distal pancreatectomies.^9)^ A thick pancreatic remnant is a recognized risk factor; accordingly, transection was performed with a reinforced stapler after gradual pre-firing compression, both of which are among the strategies used to mitigate fistula risk.^9)^ Octreotide was administered postoperatively as part of the management of the established fistula rather than as prophylaxis. A limitation of this report is that endoscopic US-guided fine-needle aspiration was not performed; nevertheless, resection was indicated by interval growth and diagnostic uncertainty irrespective of the preoperative diagnosis.

## CONCLUSIONS

Multiloculated splenic epidermoid cysts can closely mimic lymphangiomas on preoperative imaging, and integrated clinicopathological assessment—rather than any single marker—is needed to establish the correct diagnosis; in this respect, comprehensive immunohistochemistry, including D2-40, is a useful adjunct. This case also highlights that a benign splenic cyst may require distal pancreatectomy: when dense pancreatic adhesion is encountered intraoperatively, en bloc DP-S can become an unavoidable step toward safe and complete resection, and postoperative pancreatic fistula remains a relevant complication to anticipate. Careful histopathological and immunohistochemical confirmation therefore remains important for splenic cystic lesions that are radiologically indeterminate.

## DECLARATIONS

### Use of artificial intelligence (AI) tool

A generative AI tool (a large language model) was used solely to assist with English-language (grammatical) editing of this manuscript. It was not used to generate data, results, figures, or scientific content, and the authors reviewed all text and take full responsibility for the content.

### Availability of data and materials

All data generated or analyzed during this study are included in this published article.
